# Performance of Nitrogen Removal and Biofilm-Associated Microbial Community in a Compact Marine Shrimp Recirculating Aquaculture System with MBBR

**DOI:** 10.3390/microorganisms14040841

**Published:** 2026-04-08

**Authors:** Jiayan Sun, Heng Wang, Yubing Chen, Shujuan Huang, Xuejun Bi, Lihua Cheng, Xueqing Shi, Weihua Zhao, Xiaolin Zhou

**Affiliations:** College of Environmental and Municipal Engineering, Qingdao University of Technology, Qingdao 266520, China; sunjiayan@stu.qut.edu.cn (J.S.); wh20021118@163.com (H.W.); 18668939650@163.com (Y.C.); huangshujuan_2011@163.com (S.H.); xuejunb@126.com (X.B.); chenglihua666@163.com (L.C.); shixq85@163.com (X.S.); weihuazhao@qut.edu.cn (W.Z.)

**Keywords:** marine recirculating aquaculture system, moving bed biofilm reactor, biofilm carrier, microbial community, nitrogen transformation, functional prediction

## Abstract

To address ammonium nitrogen (NH_4_^+^-N) and nitrite accumulation in intensive marine shrimp aquaculture, a marine recirculating aquaculture system (RAS) for *Penaeus vannamei* centered on a moving bed biofilm reactor (MBBR) was constructed to investigate the microbial basis of nitrogen removal. The results showed that the MBBR contributed most to NH_4_^+^-N removal, demonstrating favorable nitrification potential under marine conditions (0.513 mg·L^−1^·h^−1^). The biofilm carrier formed a complete attached layer and developed a mature biofilm structure. Microbial community analysis revealed clear differentiation between the biofilm and sediment. The biofilm community was dominated by *norank_f__Caldilineaceae* (9.89%). Linear discriminant analysis effect size identified the nitrifying genus *Nitrospira* to be significantly enriched on the biofilm side (α = 0.05, linear discriminant analysis > 2.0). In addition, PICRUSt2-based functional prediction suggested a higher potential in biofilm than in sediment for ammonia oxidation and downstream nitrogen transformation, involving ammonia monooxygenase (EC:1.14.99.39), hydroxylamine dehydrogenase (EC:1.7.2.6), nitrate reductase (EC:1.7.99.4), and nitrite reductase (EC:1.7.2.1). Thus, this study provides a microbial basis and process strategy for *P. vannamei* RAS.

## 1. Introduction

Global aquaculture is undergoing a crucial transition from resource-intensive expansion to sustainable intensification. With increasing stocking densities and tightening environmental regulations, conventional aquaculture faces growing pressures from high water demand, effluent pollution, and disease risks. In this context, recirculating aquaculture systems (RASs) have emerged as an important pathway toward industrialized aquaculture because they enable high water reuse and tighter control of discharge and culture conditions [1,2,3,4].

Intensive marine shrimp cultivation practices generate elevated levels of suspended solids (SS), biochemical oxygen demand, chemical oxygen demand (COD), and nutrients in receiving waters and effluents [5]. During mariculture, aquatic animal excreta and residual bait can result in the production of nitrogenous compounds, and high-density intensive aquaculture conditions have been linked to disease risks associated with potential pathogenic bacteria [2]. Under brackish-to-marine conditions, nitrifying units can experience measurable reductions in nitrification capacity following abrupt salinity shifts, increasing the risk of nitrogen control instability during operational perturbations [6].

RASs are connected by water recirculating from culture tanks through a water treatment loop and returning to production units, enabling water reuse while relying on in-system treatment [6]. Given that RASs comprise continuous and discrete processes that are coupled and interact with each other, system performance should be understood from a process-chain perspective rather than by viewing each unit in isolation [7]. In typical RAS operations, microscreen filtration can reduce the organic/solid load entering biological treatment units and improve conditions for nitrification, thereby stabilizing downstream biological nitrogen-conversion performance [3,8,9].

Maintaining long-term water-quality stability is central to successful RAS operations, and the biological treatment unit has a decisive role by transforming toxic metabolites, such as ammonia and nitrite, thereby constraining carrying capacity and operational robustness. Among the biological treatment options, moving bed biofilm reactor (MBBR) systems are commonly used to enhance nitrification because the long residence time of the attached biofilms can facilitate the enrichment of slow-growing nitrifying bacteria [10]. In addition, MBBRs have been widely applied as a compact attached-growth technology for ammonia removal in engineered water-treatment systems, primarily through nitrification and, under suitable redox conditions, denitrification [11,12,13].

However, most studies on MBBR application in RASs remain bench or laboratory scale and are often conducted with artificial seawater or otherwise idealized, tightly controlled operating conditions [14]. Consequently, the transferability of their findings to production-relevant RASs remains incompletely resolved, particularly under coupled multi-unit operations, where solids, organics, and nitrogen are processed along the treatment chain.

At the habitat scale within RASs, suspended biofilms and sediment can host statistically distinct bacterial communities, implying potential functional differentiation relevant to nitrogen transformation pathways [15]. For shrimp RAS engineering, carrier-related traits that affect biofilm establishment (e.g., surface morphology and biofilm structural features) are considered design-relevant factors for biological nitrogen-conversion performance [16].

Therefore, in this study, we constructed a marine shrimp RAS integrating a microscreen, sand filter, protein skimmer, and an MBBR and focused on: (i) the system scale, quantifying unit-specific contributions to the removal of SS, COD, ammonium nitrogen (NH_4_^+^-N), and total phosphorus (TP), with particular emphasis on the contribution of the MBBR within the overall process chain; (ii) the micro-interface scale, characterizing biofilm development on carriers using scanning electron microscopy (SEM) and confocal laser scanning microscopy (CLSM), including structural features such as thickness and roughness; and (iii) the microbial scale, comparing bacterial community composition between MBBR suspended biofilms and system sediments using 16S rRNA gene sequencing, followed by Phylogenetic Investigation of Communities by Reconstruction of Unobserved States 2 (PICRUSt2)-based functional prediction. Together, these analyses provide system-level and mechanism-oriented evidence to support the stable and efficient application of MBBR-centered process chains in marine shrimp RASs.

## 2. Materials and Methods

### 2.1. System Description

**Figure 1:**
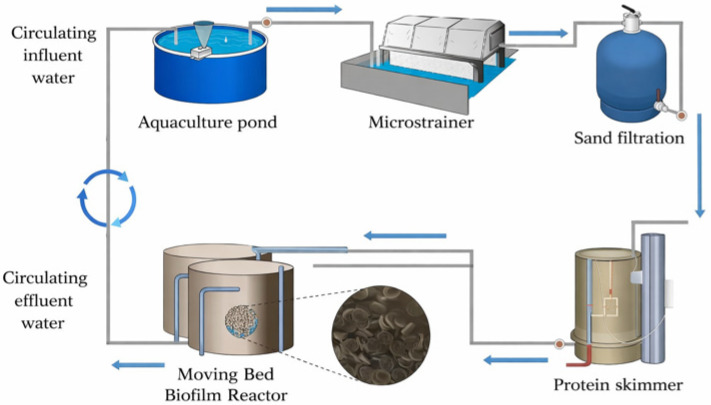
Schematic of the recirculating aquaculture system (RAS) comprising an aquaculture pond, microstrainer, sand filter, protein skimmer, and moving bed biofilm reactor (MBBR).

The RAS constructed in this study comprised an aquaculture pond, a microstrainer, a sand filter, a protein skimmer, and an MBBR (Figure 1). Water from the aquaculture pond first entered the microstrainer (60 μm mesh), where uneaten feed, feces, and other coarse SS were removed. The water then passed through the sand filter, which contained a 600-mm quartz-sand bed with an effective particle size of 0.8–1.2 mm, to further remove fine particles and colloids. Subsequently, the protein skimmer (800 mm in height and 200 mm in diameter) removed surface-active organics and part of the soluble organic matter through microbubble adsorption, thereby contributing to COD reduction.

Finally, the water entered the MBBR with an effective working volume of 200 L and a carrier-filling ratio of 30% (*v*/*v*). The carriers used were Kaldnes-type suspended media, ~10 mm in diameter with a specific surface area of 500 m^2^/m^3^. Under continuous aeration, the carriers remained in a fully mixed state, allowing biofilms to develop on their surfaces for organic degradation and nitrogen transformation. During operation, the shrimp stocking density was approximately 3000–6000 individuals m^−3^. Shrimp were fed according to growth stage following the technical specification for Litopenaeus vannamei culture in recirculating aquaculture systems. During the first 5 d of the nursery period, Artemia nauplii and shrimp flakes were supplied at 10 g·m^−3^ per feeding event and 1 g·m^−3^ per feeding event, respectively, with 8 feedings d^−1^. From day 6 to day 15, disinfected frozen adult Artemia were supplied at 20 g·m^−3^ per feeding event, with 8 feedings d^−1^. From day 16 onward, formulated feed was used as the main feed source with 6 feedings d^−1^, and the ration was adjusted to be consumed within approximately 30 min. The hydraulic retention time (HRT) was 12 h, the recirculation rate was maintained at 100%, salinity was approximately 25‰, dissolved oxygen (DO) was maintained above 6.0 mg·L^−1^, pH was maintained at 8.0–8.5, and temperature remained at 25 °C. The integrated marine RAS was operated under stable culture conditions, and water samples for process-chain analysis were collected from each treatment unit during the steady-operation period. At each sampling point, three parallel water samples were collected and analyzed independently, and the results reported as the mean ± standard deviation (SD) (n = 3).

For clarity, the integrated 200 L marine RAS was the main experimental system used for process-chain water-quality analysis, biofilm morphology characterization, bacterial community analysis, and PICRUSt2-based functional prediction, whereas the 1.0 L test was a separate bench-scale assay performed using mature biofilm carriers retrieved from the MBBR of that same system.

### 2.2. Operating Conditions

A separate bench-scale nitrification test was conducted using mature biofilm carriers retrieved from the MBBR of the integrated 200 L marine RAS after stable operation. The small-scale experiment focused solely on the MBBR. The effective working volume of this bench-scale MBBR was 1.0 L, whereas the 200 L effective working volume reported in Section 2.1 refers to the MBBR operated in the integrated RAS. The test was conducted over 90 h under ambient conditions with the water temperature maintained at 25 ± 2 °C. The reactor was continuously aerated to maintain DO at ≥6.0 mg·L^−1^, with an average of 9.3 mg·L^−1^, and the pH was maintained at 8.0–8.5. Artificial seawater was prepared as the influent with an initial COD of ~90 mg·L^−1^. Ammonium chloride, sodium carbonate, and sodium phosphate were added as nitrogen, carbon, and phosphorus sources, respectively, with the carbon:nitrogen:phosphorus molar ratio adjusted to 75:25:1. A trace element solution was also supplemented. During the experiment, COD, NH_4_^+^-N, nitrite nitrogen (NO_2_^−^-N), nitrate nitrogen (NO_3_^−^-N), TP, and pH were continuously monitored to evaluate the degradation and transformation performance of the biofilm.

During the bench-scale nitrification test, water samples at each sampling time were collected in triplicate and analyzed independently. The reported values are presented as mean ± SD (n = 3).

### 2.3. Nitrification Performance Test

To further evaluate the nitrification performance of the mature biofilm developed in the MBBR of the integrated 200 L RAS, a separate 1.0 L bench-scale test was performed using the retrieved biofilm carriers under controlled conditions. At each sampling time, three parallel water samples were collected from the reactor and analyzed independently. The samples were analyzed without filtration for COD, NH_4_^+^-N, NO_2_^−^-N, NO_3_^−^-N, TP, pH, and DO. All results were expressed as the mean ± SD (n = 3).

COD was determined using the dichromate digestion method: 0.08–0.09 g of mercuric sulfate, 0.75 mL of potassium dichromate solution, and 2.25 mL of silver sulfate–sulfuric acid solution were added to the COD tube, followed by digestion at 150 °C for 2 h and direct spectrophotometric measurement. NH_4_^+^-N was analyzed by the Nessler’s reagent colorimetric method, where 1 mL of buffer solution and 0.15 mL of color reagent were added to the sample, and absorbance was measured after 10 min. NO_2_^−^-N was determined by the N-(1-naphthyl)-ethylenediamine spectrophotometric method, with color development for 20 min before measurement. NO_3_^−^-N was determined by ultraviolet spectrophotometry after adding 1 mL of hydrochloric acid and 0.7 mL of phenol solution, with reaction for 20–30 min, followed by measurement in a 5-mm cuvette. TP was determined by the molybdenum–antimony anti-colorimetric method: samples were digested with potassium persulfate, then ammonium molybdate and ascorbic acid were added under acidic conditions to form a blue complex, which was measured spectrophotometrically. pH and DO were measured in situ using portable meters.

**Figure 2:**
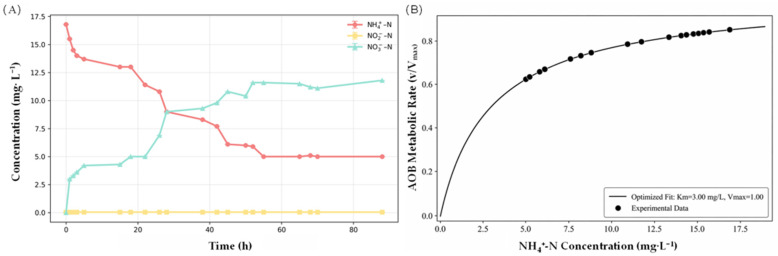
Concentration variations of ammonium nitrogen (NH_4_^+^-N), nitrite nitrogen (NO_2_^−^-N), and nitrate nitrogen (NO_3_^−^-N) during the nitrification performance test for the suspended biofilms (**A**), and the Michaelis–Menten fit for the apparent ammonia oxidation rate as a function of NH_4_^+^-N concentration (**B**). Data in panel (**A**) are presented as the mean ± SD (n = 3), and the error bars represent SD. The kinetic fitting in panel (**B**) was performed using the mean NH_4_^+^-N concentrations from triplicate measurements.

For the kinetic analysis in Figure 2B, the apparent ammonia oxidation rate (v, mg NH_4_^+^-N·L^−1^·h^−1^) was calculated using the mean NH_4_^+^-N concentrations obtained from triplicate measurements at two consecutive sampling points divided by the corresponding time interval. The relationship between the apparent rate (v) and NH_4_^+^-N concentration (S, mg·L^−1^) was fitted using the Michaelis–Menten equation, v = Vmax·S (K_m_ + S)^−1^, where Vmax represents the maximum apparent ammonia oxidation rate and K_m_ represents the half-saturation constant. Nonlinear regression was performed using OriginPro 2024 (OriginLab Corporation, Northampton, MA, USA), and the fitted parameters were obtained from the best-fit model.

### 2.4. Biofilm Morphology

To investigate the morphological differences of suspended carriers before and after biofilm development, SEM and CLSM were used. The biofilm carriers used for SEM and CLSM observation were collected directly from the MBBR of the integrated 200 L marine RAS after stable operation. For SEM preparation, carriers were gently rinsed with ultrapure water to remove loosely attached impurities, fixed in 2.5% glutaraldehyde solution for 12 h, dehydrated through a graded ethanol series (30%, 50%, 70%, 90%, and 100%), dried with a critical point dryer, sputter coated with gold, and then observed using a ZEISS SIGMA 500 scanning electron microscope (Oberkochen, Germany). For CLSM, carriers were stained with SYTO 9 and propidium iodide for 20 min, rinsed with PBS, and examined under a Leica TCS SP8 confocal laser scanning microscope (Wetzlar, Germany). CLSM images were reconstructed in 3D using image analysis software to quantify surface roughness and biofilm thickness.

### 2.5. Bacterial Community Analysis

Biofilm and sediment samples used for microbial community analysis were collected directly from the integrated 200 L marine RAS after stable operation. Specifically, three random biofilm samples were obtained from the carrier-associated biofilm, and three random sediment samples were collected from the deposits in the biological chamber. All six samples were sent to Majorbio Bio-Pharm Technology Co., Ltd. (Shanghai, China) for genomic DNA extraction and high-throughput sequencing according to standard procedures. The hypervariable V3–V4 region of the bacterial 16S rRNA gene was amplified using the primer pair 338F/806R. The sequencing data were processed following the standard workflow of Majorbio Bio-Pharm Technology Co., Ltd. Operational taxonomic unit representative sequences were taxonomically assigned against the Silva138 database using a confidence threshold of 0.7. Most downstream statistical analyses were performed on the Majorbio Cloud Platform. Community composition was summarized mainly at the genus level, and the relative abundance of selected nitrification-related taxa was examined at the phylum level. Beta diversity was analyzed based on Bray–Curtis dissimilarity using non-metric multidimensional scaling (NMDS), and differentially enriched taxa between the biofilm and sediment samples were identified by linear discriminant analysis effect size (LEfSe) with a linear discriminant analysis (LDA) cutoff of 2.0.

### 2.6. Potential Functions Analysis

Potential microbial functions were predicted using PICRUSt2 based on the 16S rRNA gene sequencing data. Given the continuous refinement of reference genome databases, PICRUSt2 has been reported to provide improved functional prediction accuracy relative to earlier versions. Benchmarking against paired 16S rRNA gene and shotgun metagenomic datasets has shown that PICRUSt2 can provide robust metagenome inference, although its outputs should still be interpreted as predicted functional potential rather than direct functional verification [17]. Predicted functions related to nitrogen metabolism were screened with reference to Kyoto Encyclopedia of Genes and Genomes (KEGG) map00910. The prediction outputs were summarized at the levels of enzyme commission (EC), KEGG ortholog, module, and pathway to characterize potential microbial functions associated with ammonia oxidation, nitrate/nitrite transformation, and denitrification.

## 3. Results and Discussion

### 3.1. Process Overview

The microstrainer represented the first monitored stage of the RAS and provided the baseline for evaluating the subsequent treatment units. At this stage, NH_4_^+^-N, SS, COD, and TP were 1.71 ± 0.03 mg·L^−1^, 80.00 ± 1.35 mg·L^−1^, 75.00 ± 1.59 mg·L^−1^, and 0.90 ± 0.02 mg·L^−1^, respectively, while pH remained within 8.0–8.5. Given that water-quality comparisons among units were conducted from the microscreened water onward, the microstrainer is presented here as the initial process-chain reference point rather than as a separately quantified removal step.

Relative to the microstrainer stage, the sand filter acted as the main physical barrier for particulate matter. SS decreased from 80.00 ± 1.35 mg·L^−1^ to 35.00 ± 0.79 mg·L^−1^ (56.3% removal), COD decreased from 75.00 ± 1.59 mg·L^−1^ to 60.00 ± 1.39 mg·L^−1^ (20.0% removal), and TP decreased from 0.90 ± 0.02 mg·L^−1^ to 0.65 ± 0.02 mg·L^−1^ (27.8% removal). In contrast, NH_4_^+^-N increased slightly from 1.71 ± 0.03 mg·L^−1^ to 1.96 ± 0.03 mg·L^−1^ (14.6% increase). These results indicate that sand filtration mainly targeted nondissolved pollutants, effectively removing SS as well as particle-bound COD and phosphorus, while showing limited direct removal of dissolved ammonia.

The protein skimmer showed a different pattern. Relative to the sand-filter effluent, SS decreased from 35.00 ± 0.79 mg·L^−1^ to 32.00 ± 0.85 mg·L^−1^ (8.6% removal), COD decreased from 60.00 ± 1.39 mg·L^−1^ to 25.00 ± 1.23 mg·L^−1^ (58.3% removal), and TP decreased from 0.65 ± 0.02 mg·L^−1^ to 0.58 ± 0.02 mg·L^−1^ (10.8% removal). In contrast, NH_4_^+^-N changed only slightly, from 1.96 ± 0.03 mg·L^−1^ to 1.99 ± 0.04 mg·L^−1^ (1.5% increase). This pattern is consistent with the operating mechanism of protein skimming, in which bubble–interface adsorption preferentially removes surface-active matter and colloidal organic matter, whereas dissolved inorganic nitrogen is less affected.

The MBBR showed the strongest ammonia conversion and substantial residual COD removal among the treatment units. From the skimmer effluent to the MBBR effluent, NH_4_^+^-N decreased from 1.99 ± 0.04 mg·L^−1^ to 0.66 ± 0.03 mg·L^−1^ (66.8% removal), whereas COD decreased from 25.00 ± 1.23 mg·L^−1^ to 10.00 ± 0.69 mg·L^−1^ (60.0% removal). TP further decreased slightly from 0.58 ± 0.02 mg·L^−1^ to 0.56 ± 0.02 mg·L^−1^ (3.4% removal). Meanwhile, SS increased from 32.00 ± 0.85 mg·L^−1^ to 40.00 ± 1.15 mg·L^−1^ (25.0% increase), possibly because of biofilm detachment. During passage through the MBBR, NH_4_^+^-N exhibited a transient increase within the reactor before decreasing markedly in the final circulating effluent, which might reflect internal mixing together with progressive ammonia conversion within the biological unit. Given that ammonia oxidation is primarily an autotrophic process, the observed residual COD removal was likely associated with aerobic biodegradation, biomass assimilation, and solids retention, whereas the decline in NH_4_^+^-N was primarily attributable to nitrification under the high DO conditions of the system (9.3 mg·L^−1^).

The process-chain comparison revealed a clear division of labor among the treatment units. The microstrainer provided the initial pretreatment baseline, the sand filter mainly removed suspended and particle-associated pollutants, the protein skimmer contributed the greatest physicochemical reduction in COD, and the MBBR served as the core biological unit for ammonia conversion. This process-level division of labor is broadly consistent with previous RAS studies showing that mechanical solids-removal units primarily reduce particulate loads, foam fractionation improves the control of dissolved and fine organic matter, and biofilters provide the main nitrification capacity [9,18,19,20].

The overall removal performance of each unit operation is summarized in Table 1.

### 3.2. Bench-Scale Verification of Nitrification Performance of the Same-Source MBBR Biofilm

Using mature biofilm carriers retrieved from the MBBR of the integrated 200 L RAS, a separate bench-scale test was conducted to verify the nitrification behavior of the same-source biofilm under controlled conditions. During this test, the concentration variations of NH_4_^+^-N, NO_2_^−^-N, and NO_3_^−^-N were continuously monitored over time. NH_4_^+^-N exhibited a three-phase decline, decreasing from 16.742 ± 0.016 mg·L^−1^ at 0 h to 5.006 ± 0.010 mg·L^−1^ at 89 h. Simultaneously, NO_3_^−^-N increased from 0.150 ± 0.005 mg·L^−1^ to 11.802 ± 0.020 mg·L^−1^, whereas NO_2_^−^-N remained at relatively low levels (0.001–0.038 mg·L^−1^). These trends indicate progressive oxidation of ammonium, with efficient nitrite conversion and subsequent nitrate accumulation. However, because total nitrogen balance and gaseous nitrogen measurements were not performed in this study, the exact nitrogen loss pathway could not be confirmed. Possible sinks may include microbial assimilation, retention in biofilm/sludge, and limited ammonia volatilization under aerated alkaline conditions.

During the initial 6 h, the NH_4_^+^-N concentrations rapidly decreased from 16.742 ± 0.016 mg·L^−1^ to 13.662 ± 0.016 mg·L^−1^, corresponding to a removal rate of 0.513 mg·L^−1^·h^−1^. Meanwhile, NO_3_^−^-N increased to 4.450 ± 0.010 mg·L^−1^ at 6 h. NO_2_^−^-N concentrations remained minimal (<0.01 mg·L^−1^), indicating efficient conversion to NO_3_^−^-N. Michaelis–Menten fitting of the apparent ammonia oxidation rate against NH_4_^+^-N concentration yielded a half-saturation constant (Km) of 3.00 mg·L^−1^ (Figure 2B). The high NH_4_^+^-N removal rate is consistent with strong ammonia oxidation activity under high-substrate conditions because the NH_4_^+^-N concentrations were well above the half-saturation constant (K_m_ = 3.00 mg·L^−1^) [21]. In addition, the high DO (>9.3 mg·L^−1^) likely favored complete nitrification and helped prevent NO_2_^−^-N accumulation.

Between 6 and 42 h, NH_4_^+^-N concentrations decreased from 13.662 mg·L^−1^ to 7.703 mg·L^−1^, with a significantly reduced removal rate of 0.166 mg·L^−1^·h^−1^, a 67.6% decline compared to the initial phase. NO_3_^−^-N continued to accumulate but at a reduced rate of 0.150 mg·L^−1^·h^−1^, reaching 9.863 mg·L^−1^ at 42 h. During this phase, NO_2_^−^-N exhibited slight fluctuations, peaking at 0.038 mg·L^−1^ at 18 h before declining to 0.012 mg·L^−1^ at 42 h. This temporary increase suggests a transient imbalance between ammonia-oxidizing bacteria and nitrite-oxidizing bacteria (NOB) activities, possibly because of a lag in NOB adaptation to reduced NH_4_^+^-N availability [22].

Between 42 and 90 h, NH_4_^+^-N concentrations remained between 4.972–5.006 mg·L^−1^, with a notably reduced removal rate of 0.057 mg·L^−1^·h^−1^. Concurrently, NO_3_^−^-N increased to 11.802 mg·L^−1^ at a reduced accumulation rate of 0.041 mg·L^−1^·h^−1^, suggesting a plateauing trend. This pattern might reflect reduced apparent ammonia oxidation activity under prolonged low NH_4_^+^-N conditions [23]. In addition, oxygen diffusion limitations within the biofilm matrix could also have contributed to lower oxidation efficiency, because thicker biofilms can generate internal microenvironments with reduced substrate availability in deeper layers.

These results indicate that the nitrification process was highly efficient during the early phase, transitioning into a substrate-limited state in the intermediate phase, and stabilizing at a lower metabolic rate during the late phase, highlighting the dynamic nature of biofilm-based nitrification systems.

### 3.3. Biofilm Morphology

**Figure 3:**
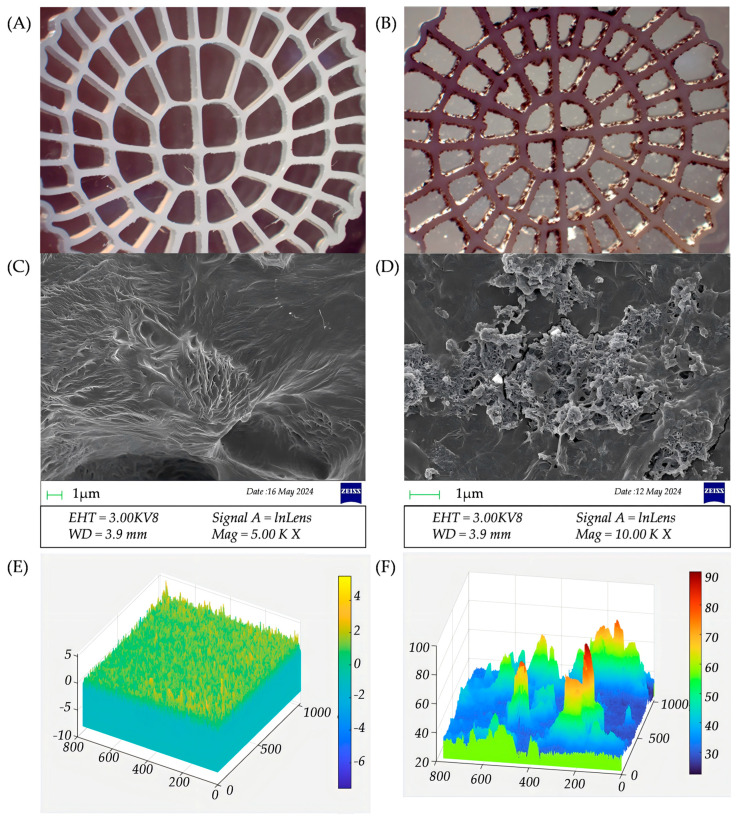
Morphology of the (**A**) new and (**B**) biofilm carriers observed by electron microscopy, by scanning electron microscopy (SEM) for (**C**) new and (**D**) biofilm carriers, and by confocal laser scanning microscopy (CLSM) for (**E**) new and (**F**) biofilm carriers.

To analyze the biofilm morphology, a comparison was made between the new carrier (before biofilm attachment) and the biofilm carrier (post-biofilm attachment). Microscopy techniques, including electron microscopy, SEM, and CLSM, were used to examine the carrier morphology and biofilm development (Figure 3). Electron microscopy images revealed no obvious microbial adhesion on the surface of the new carrier (Figure 3A), whereas the biofilm carrier (Figure 3B) exhibited a well-developed attached structure.

SEM imaging at 10,000× magnification showed that the new carrier (Figure 3C) had a textured surface with intricate microstructures, which likely provided favorable attachment sites for microbial colonization. Such surface roughness can increase the contact area between microorganisms and the carrier, thereby enhancing interfacial interactions and favoring biofilm establishment [24]. In contrast, the biofilm carrier (Figure 3D) exhibited evident microbial adhesion, with a dense microbial layer forming a visible biofilm. To further investigate the structural differences, two areas were randomly selected from each carrier for CLSM imaging.

The CLSM images (Figure 3E,F) further highlighted the structural difference between the two carriers, with clear microbial adhesion observed on the biofilm carrier surface (Figure 3F). CLSM data indicated that the average surface roughness of the new carrier was 0.538 µm and 0.687 µm in the selected regions, with maximum heights of 10.757 µm and 13.094 µm. For the biofilm carrier, the arithmetic average heights were 3.400 µm and 5.661 µm, with maximum heights of 44.867 µm and 69.952 µm. These differences indicate substantial biofilm development on the carrier surface. Furthermore, the inherent roughness of the new carrier likely provided initial attachment points for subsequent biofilm formation and retention [25].

SEM and CLSM observations further indicated that the biofilm carrier developed a thicker and more heterogeneous attached layer compared with the new carrier. This structural differentiation could imply physicochemical heterogeneity within the attached matrix. However, because dissolved oxygen microprofiles and direct denitrification measurements were not obtained in the present study, these observations should be regarded only as indirect structural evidence and not as direct proof of simultaneous nitrification and denitrification (SND) [26].

In addition, surface roughness also has an important role in biofilm formation by directly influencing microbial adhesion and biofilm stability. Lago et al. [27] reported that increased surface roughness enhances microbial attachment and favors the formation of stronger and more resilient biofilms. Furthermore, research in *Electrochimica Acta* [28] highlighted that structured surfaces can enhance surface-associated biological activity, further supporting the functional importance of surface architecture.

These findings collectively suggest that biofilm thickness and surface roughness are important structural factors associated with biofilm stability, carrier retention, and treatment performance.

### 3.4. Bacterial Community

**Figure 4:**
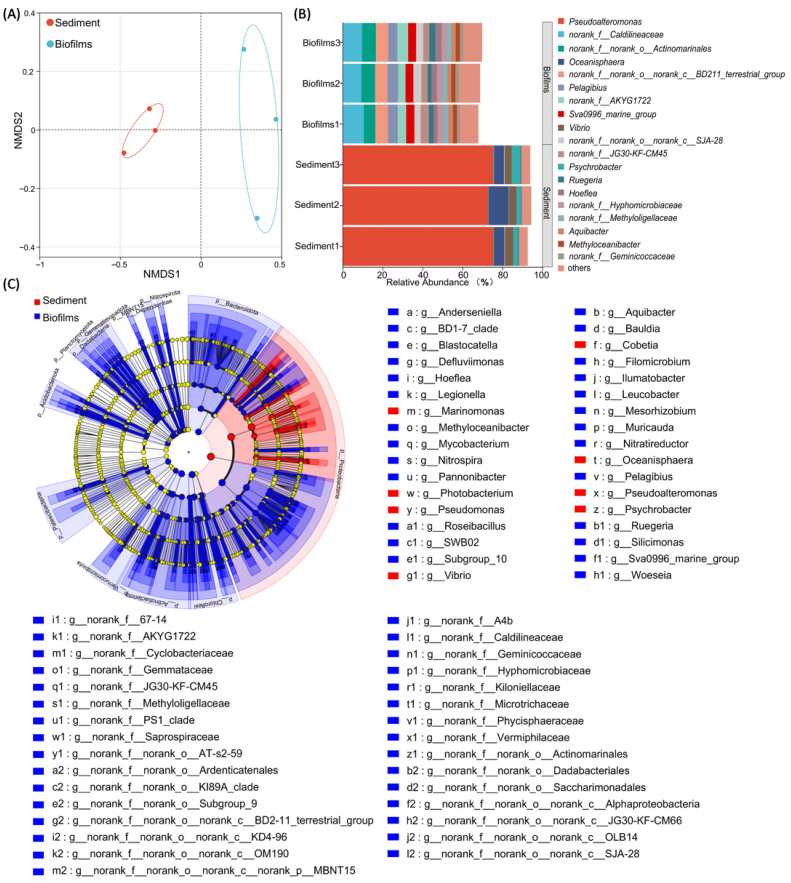
Genus-level community differentiation and discriminant taxa in the biofilm and sediment samples. (**A**) Non-metric multidimensional scaling (NMDS) ordination based on Bray–Curtis dissimilarity. Samples formed two habitat-defined clusters with minimal overlap (stress = 0.001, rounded). (**B**) Stacked bar plots of genus-level relative abundance for the top-20 genera, with ‘others’ representing the remaining genera; samples are grouped by habitat to illustrate habitat-specific community composition. (**C**) Linear discriminant analysis effect size (LEfSe) cladogram showing discriminant taxa from phylum to genus. Red indicates taxa enriched on the sediment side, and blue indicates taxa enriched on the biofilm side. Node size is proportional to relative abundance, and significant discriminant taxa are listed on the right. LEfSe was performed with α = 0.05 and a linear discriminant analysis (LDA) cutoff of 2.0.

Genus-level NMDS based on Bray–Curtis dissimilarity clearly separated the biofilm and sediment samples (Figure 4A). The 2D NMDS solution showed an excellent fit (stress = 0.001, rounded). Sediment samples clustered on the negative side of NMDS1, whereas biofilm samples clustered on the positive side, with minimal overlap between the two groups.

Consistent with the NMDS separation, stacked bar plots of the top-20 genera revealed clear group-specific compositional differences between the biofilm and sediment samples (Figure 4B). Within the biofilm set, *norank_f__Caldilineaceae* ranked first in mean relative abundance, accounting for 9.89% of the community. LEfSe further identified *norank_f__Caldilineaceae* as a discriminant taxon enriched in the biofilm side (Figure 4C). Recent studies identified *norank_f__Caldilineaceae* as a denitrification-related taxon in wastewater systems, and its higher abundance has been associated with enhanced denitrification performance, suggesting a potential role in nitrate/nitrite transformation under saline conditions [29]. *Pelagibius* showed a similar distribution pattern, with a mean relative abundance of 4.91% in the biofilm. *Pelagibius* has been reported to be a marine, strictly aerobic taxon, and its occurrence in the present study might reflect adaptation to saline environments [30].

Within the sediment set, *Pseudoalteromonas* ranked first in mean relative abundance, accounting for 74.6% of the community (Figure 4B). LEfSe likewise identified *Pseudoalteromonas* as a discriminant taxon enriched in the sediment group (Figure 4C). Recent studies suggest that beyond its roles in algal-polysaccharide turnover and secondary-metabolite production, *Pseudoalteromonas* also provides labile organic substrates and matrix-associated components that could support heterotrophic denitrification under saline conditions, thereby contributing to nitrogen transformation [22,31]. *Oceanisphaera* ranked second in the sediment group, with a mean relative abundance of 6.64% (Figure 4B), and might also contribute to nitrate reduction under saline and micro-oxic conditions [32]. *Vibrio* ranked third in the sediment group, with a mean relative abundance of 3.71% (Figure 4B); this is an important marine chitin-degrading taxon that can utilize chitin-derived substrates as carbon and nitrogen sources, thereby contributing to organic matter turnover and nutrient cycling in saline environments [33].

Moreover, LEfSe identified the nitrifying genus *Nitrospira* to be significantly enriched on the biofilm side (α = 0.05, LDA > 2.0). At the phylum level, *Nitrospirota* accounted for 0.39%, further supporting the presence of nitrification-related bacteria in the biofilm community [34,35]. These taxonomic differences suggest ecological differentiation between the biofilm and sediment compartments and provide context for the functional prediction results presented below.

### 3.5. Potential Functions for Nitrogen Removal

**Figure 5:**
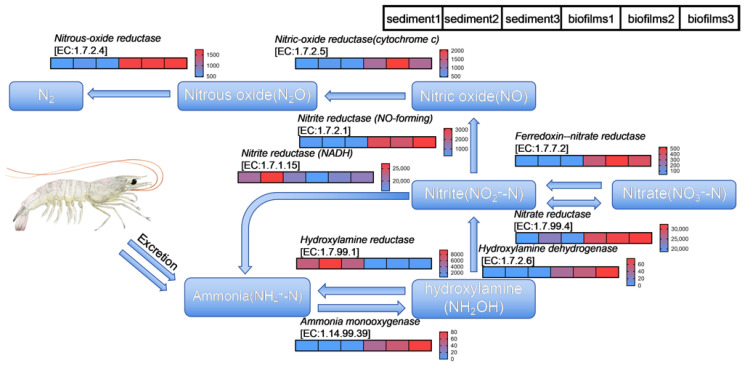
Heatmap for sequence numbers of the potential functions for nitrogen removal of the suspended biofilms and sediment in the RAS.

The potential nitrogen metabolism functions of the suspended biofilm and sediment in this system are shown in Figure 5. These functional predictions were generally consistent with the community composition patterns described above. Functional prediction revealed that microorganisms in the biofilm exhibit stronger nitrification potential compared with those in the sediment, which is consistent with the enrichment of *Nitrospira* on the biofilm side. Microorganisms in the biofilm may initiate the ammonia oxidation process by converting ammonia released during *P. vannamei* culture into hydroxylamine via the key enzyme ammonia monooxygenase [EC:1.14.99.39] [36]. The average sequence number of this enzyme in the biofilm was 61, compared with 0.33 in the sediment. Subsequently, hydroxylamine may be further transformed through hydroxylamine dehydrogenase [EC:1.7.2.6] and nitrate reductase [EC:1.7.99.4] [37,38], with average sequence numbers of 55.50 and 31,240.13 in the biofilm, respectively.

PICRUSt2 prediction suggested the potential for nitrate and nitrite reduction to nitric oxide (NO), associated with ferredoxin-nitrate reductase [EC:1.7.7.2] and nitrite reductase (NO-forming) [EC:1.7.2.1] [39]. Finally, NO is reduced to nitrous oxide and further to nitrogen gas, which is released into the atmosphere via nitric-oxide reductase (cytochrome c) [EC:1.7.2.5] and nitrous-oxide reductase [EC:1.7.2.4]. However, because these results were derived from PICRUSt2-based functional prediction, they should be interpreted as potential pathways rather than as direct evidence of gaseous nitrogen release. The average sequence numbers for these enzymes in the biofilm were 448.55, 2770.11, 1549.41, and 1711.94. Concurrently, the predicted sequence numbers for hydroxylamine reductase [EC:1.7.99.1] and nitrite reductase (NADH) [EC:1.7.1.15] in the sediment were significantly higher than those in the biofilm, with average sequence numbers of 713.52 and 22,653.33 in the sediment, respectively. This pattern may indicate relatively stronger reductive nitrogen turnover and possible ammonium regeneration potential in the sediment compartment. These results should be interpreted as inferred functional potential rather than direct evidence of in situ gene abundance or enzyme activity.

Given that the culture of *P. vannamei* continuously releases ammonia nitrogen into the system, excessive ammonia accumulation in the culture water can be toxic to shrimp. Based on PICRUSt2 prediction, microorganisms in the biofilm may have the potential to mediate ammonia oxidation and downstream nitrate/nitrite reduction pathways. Taken together with the process-chain results and community composition, these predictions are consistent with the MBBR being the main unit associated with ammonia nitrogen removal in the system. During *P. vannamei* culture, the MBBR could help alleviate ammonia toxicity risks to shrimp.

## 4. Conclusions

This work demonstrates that a compact marine RAS for *P. vannamei* can achieve stable water-quality control through a clear division of labor among unit processes. Sand filtration mainly intercepted SS and particulate-associated pollutants, whereas protein skimming provided the dominant physicochemical removal of surface-active and colloidal organic matter. The MBBR functioned as the principal biological barrier for reactive nitrogen, delivering the largest NH_4_^+^-N decrease and sustaining efficient nitrification under high salinity and high DO. The observed three-phase nitrification trajectory (rapid initial oxidation followed by substrate-limited deceleration and a low-rate plateau), together with negligible nitrite accumulation, is consistent with efficient coupling between ammonium- and nitrite-oxidizing guilds within the carrier-associated biofilm.

Mechanistically, carrier–biofilm engineering appears to be a practical means of shaping nitrogen transformation in compact saline systems. SEM/CLSM showed that biofilm maturation markedly increased the apparent surface roughness and thickness, which may suggest greater structural heterogeneity within the attached matrix. Consistent with this structural differentiation, 16S-based community profiling indicated clear compositional differentiation between the suspended biofilms and surrounding sediments. Importantly, PICRUSt2-based functional inference with KEGG annotation further suggested that biofilm communities harbored higher predicted potentials for key steps of ammonia oxidation and downstream nitrogen transformation compared with sediments, whereas sediments showed signatures more consistent with reductive nitrogen turnover and ammonium regeneration. These inferences represent predicted functional potential rather than direct validation of gene abundance or activity. Together, these results support a conceptual model in which the MBBR likely represents the principal biological compartment associated with shrimp-derived NH_4_^+^-N removal, while sediments may serve as a secondary transformation zone contributing to internal reactive nitrogen recycling. Future work combining metagenomic or reverse transcription quantitative polymerase chain reaction (RT-qPCR) validation of nitrogen-cycling genes with direct measurements of microscale redox profiles will be needed to better quantify potential anoxic microzones and to relate predicted functional capacity to in situ process rates under production-scale loading.

## Figures and Tables

**Table 1:** Typical concentration profiles of SS, COD, NH_4_^+^-N, TP, and pH across treatment units.

Treatment Unit	NH_4_^+^-N (mg·L^−1^)	SS (mg·L^−1^)	COD (mg·L^−1^)	TP (mg·L^−1^)	pH
Microstrainer	1.71 ± 0.03	80.00 ± 1.35	75.00 ± 1.59	0.90 ± 0.02	8–8.5
Sand filtration	1.96 ± 0.03	35.00 ± 0.79	60.00 ± 1.39	0.65 ± 0.02	8–8.5
Protein skimmer	1.99 ± 0.04	32.00 ± 0.85	25.00 ± 1.23	0.58 ± 0.02	8–8.5
Moving bed biofilm reactor	0.66 ± 0.03	40.00 ± 1.15	10.00 ± 0.69	0.56 ± 0.02	8–8.5

## Data Availability

The original contributions presented in this study are included in the article. Further inquiries can be directed to the corresponding author.
